# Knorr-Rabe partial reduction of pyrroles: Application to the synthesis of indolizidine alkaloids

**DOI:** 10.1186/1860-5397-4-3

**Published:** 2008-01-15

**Authors:** Brendon S Gourlay, John H Ryan, Jason A Smith

**Affiliations:** 1 School of Chemistry, University of Tasmania, Hobart, Australia; 2 CSIRO Division of Molecular and Health Technologies, Bag 10, Clayton South, Victoria, Australia

## Abstract

**Background:**

The Birch reduction of electron rich pyrroles does not occur readily. However, dissolving metal reduction with zinc under acidic conditions gives 3-pyrrolines (2,5-dihydropyrroles) in reasonable yield. This dissolving metal reduction was first reported by Knorr and Rabe in 1901 but since then has only been reported for the reduction of electron rich pyrroles.

**Results:**

The partial reduction of bicyclic α-ketopyrrole derivatives has been performed under dissolving metal conditions with zinc and hydrochloric acid to give excellent yields of hexahydroindolizidines. This reduction method has been utilised for the diastereoselective synthesis of 5-alkylindolizidines and the stereoselectivity obtained is opposite to that of catalytic hydrogenation.

**Conclusion:**

An efficient stereoselective synthesis of indolizidine alkaloids has been developed from α-ketopyrrole intermediates using a modified version of Knorr and Rabe's pyrrole reduction.

## Background

**Scheme 1:**
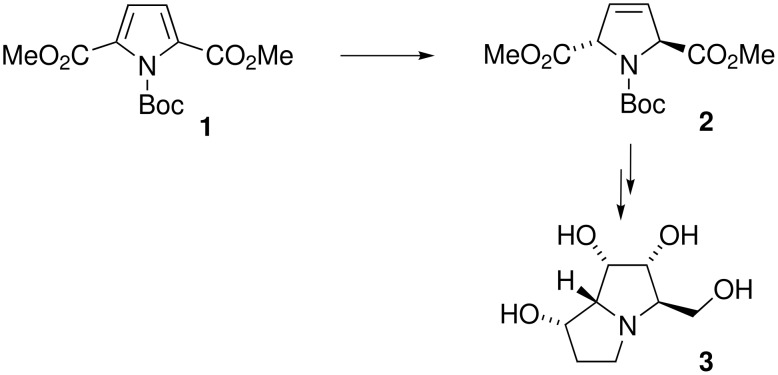
Donohoe's approach to (±)-1-epiaustraline utilising a modified Birch reduction.

The Birch reaction for the dearomatisation of aromatic substrates is an extremely practical and important tool for synthetic chemists and is used widely as a key step for the synthesis of natural products and molecules of biological interest [1]. However, the partial reduction of pyrrole is difficult as the high electron density of these aromatic heterocycles inhibits the addition of an electron, the first step of a Birch reaction [2]. Donohoe has shown that the partial reduction of pyrroles is possible but this process generally requires the presence of at least two electron withdrawing groups that reduce the electron density of the heterocycle such that reasonable yields of the 3-pyrrolines are obtained [3]. This method was recently exploited for the elegant synthesis of the pyrrolidine alkaloid (±)-1-epiaustraline (**3**) (Scheme 1) [4].

**Scheme 2:**
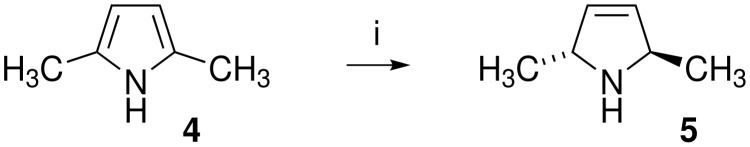
Reaction conditions i) Zn, HCl (aq).

During our studies towards the synthesis of indolizidine alkaloids we required bicyclic 3-pyrrolines and chose to explore accessing these intermediates *via* partial reduction of the corresponding pyrrole derivatives. These substrates were far more electron rich than those of Donohoe and thus not amenable to Birch reduction methodology. Therefore, we turned to an underutilised reaction that was reported by Knorr and Rabe [5] in 1901 and has only been reported a handful of times since [6–9]. The method employs powdered zinc in an acid media to give 3-pyrrolines, presumably by protonation of the pyrrole to give an iminium ion which is then reduced. It has been shown that reaction of 2,5-dialkylpyrroles gives predominantly the *trans* 3-pyrroline isomer (Scheme 2) [7–9].

## Results and Discussion

**Scheme 3:**
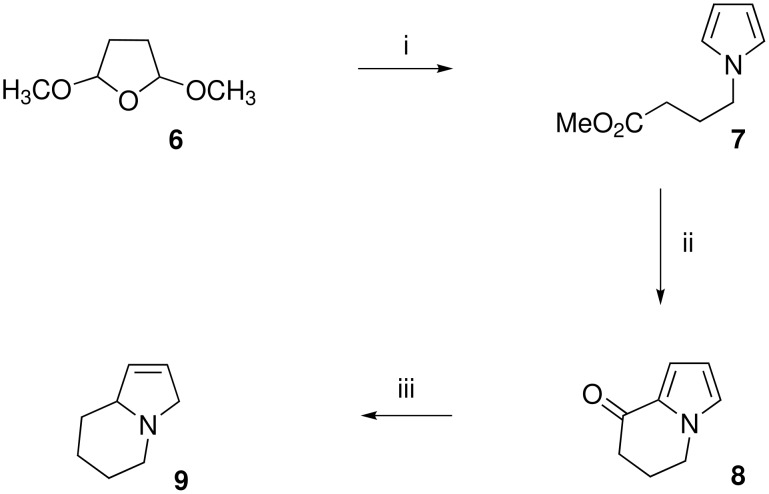
Reaction conditions: i) ref. [10] ii) ref. [11] iii) Zn, conc. HCl_(aq)_.

The synthetic plan that we adopted was to construct a bicyclic pyrrole derivative by exploiting the natural reactivity of pyrrole and then to partially reduce the heterocyclic core (Scheme 3). The synthesis started with formation of the γ-pyrrolic ester **7** in high yield using an improved Clauson-Kaas synthesis [10], followed by boron tribromide mediated cyclisation to give the known bicyclic ketone **8** [11]. Upon subjection of this α-ketopyrrole **8** to the modified conditions reported by Andrews and McElvain (slow addition of HCl to the substrate and Zn at 0–10 °C) [5,9] we observed no reaction and starting material was returned. However, when zinc and concentrated HCl were added in small portions to a hot solution of the α-ketopyrrole in methanol over ~10 minutes the starting material was consumed to give the hexahydroindolizidine **9** as the only observable product in ~80% yield. The chemoselectivity using these modified conditions is noteworthy while the carbonyl group is fully reduced the pyrrole group is selectively and partially reduced to the 3-pyrroline. This result was confirmed by comparison of the spectral data with that reported by Huxtable who prepared **9** as an intermediate in the synthesis of lentiginosine [12].

For the partial reduction of electron rich pyrroles reported previously, over reduction to give pyrrolidines is a problematic side-reaction. For example, Andrews and McElvain kept the reaction temperature below 10 °C to limit pyrrolidine formation. Under our conditions, starting with the α-ketopyrrole, there was no indication of pyrrolidine formation. The loss of the keto group means that the product is the same as that that would be obtained by reduction of the parent bicyclic pyrrole **13**. The reduction of the carbonyl group resembles that of a Clemmensen reduction; however, amalgamated zinc is required for Clemmensen reaction [13].

**Scheme 4:**
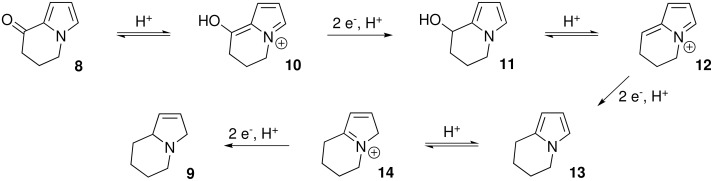
Potential mechanism for α-ketopyrrole reduction..

There are several possible mechanisms for this transformation, however, we propose the first step involves protonation of the carbonyl group to give a conjugated iminium ion **10** (Scheme 4). This species would undergo a two-electron reduction process, with associated protonation to give the α-hydroxy pyrrole **11**. Acid-promoted dehydration of **11** would afford a second iminium ion **12** which could undergo further reduction and protonation to give pyrrole **13**. The pyrrole could then be protonated to give a third iminium ion **14** and reduction would then give rise to the product **9**.

**Scheme 5:**
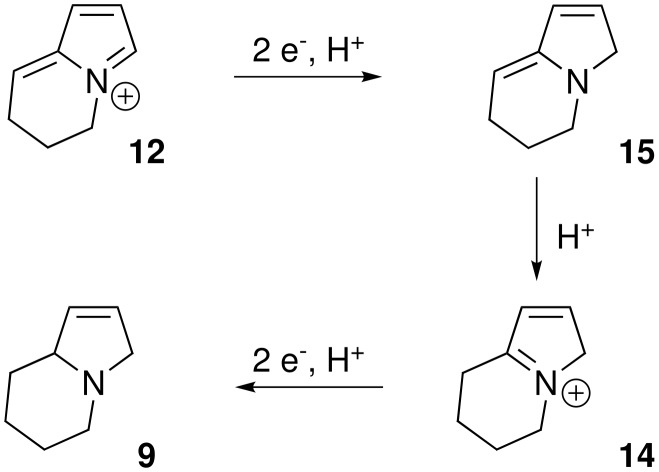
Alternative reduction pathway.

Our reaction conditions are much harsher than those previously reported, and yet we do not see pyrrolidine products and this suggests that an alternative pathway is in operation. One possibility is that the intermediate **12** could undergo reduction to give the final product directly without the formation of the pyrrole intermediate **13** (Scheme 5).

To test these hypotheses we reduced the ketone **8** with NaBH_4_ to give the unstable α-hydroxy pyrrole **11** which was then immediately subjected to the reduction conditions. The same result was obtained giving the 3-pyrroline **9** which lends support to the suggestion that **11** is an intermediate in the reaction. When pyrrole **13** was reacted under the same conditions **9** was formed but the ^1^H NMR spectrum also showed some starting material remained. The fact that the pyrrole **13** was not observed in the reduction products from α-ketopyrrole **8** lends the support to the suggestion of an alternative pathway. At the present time the intermediacy of **13** cannot be ruled out for the reduction of ketone **8** and alcohol **11**.

**Scheme 6:**
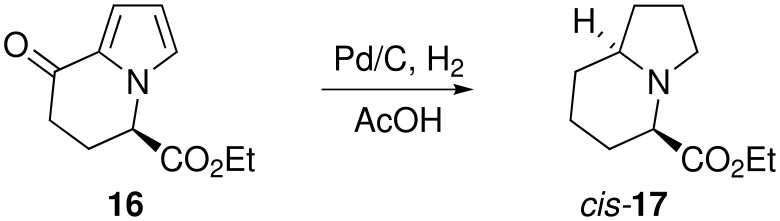
Catalytic hydrogenation.

Due to the facile and rapid reaction of the α-ketopyrrole **8** we explored the potential tandem α-ketopyrrole reduction/catalytic hydrogenation as an alternative to catalytic hydrogenation. The catalytic hydrogenation of 5-substituted tetrahydroindolizidines proceeds with high diastereoselectivity [14–15] and has also been exploited for the synthesis of numerous indolizidine alkaloids [16–17]. The presence of a substituent at C-5 directs the hydrogenation at C-8a from the opposite, least hindered face, to give the *cis* derivative (Scheme 6).

**Scheme 7:**
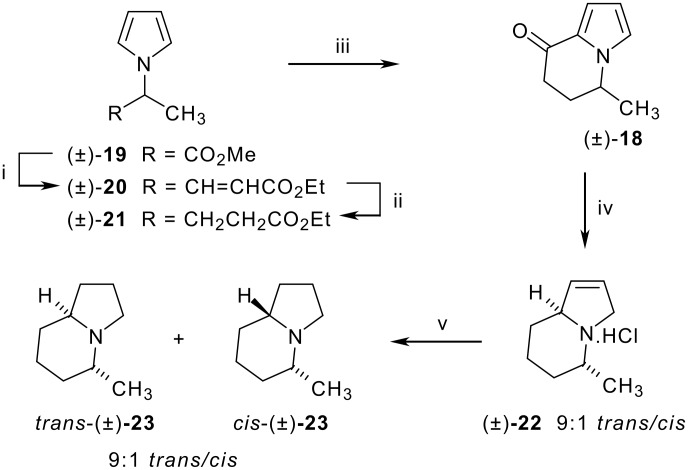
Reaction conditions: i) DIBAL-H, CH_2_Cl_2_, -78 °C, 1 h then triethylphosphonoacetate, NaH, THF, −78 °C – rt ii) H_2_ (40psi), Pd/C, EtOH iii) BBr_3_, CH_2_Cl_2_, 0 °C, 10 min iv) Zn, conc. HCl(aq) v) H_2_ (40 psi), Pd/C, EtOH, 2M HCl.

We were interested in the stereochemical outcome for C-8a using the modified Knorr-Rabe zinc reduction and synthesised the known 5-methyl derivative **18** (Scheme 7) as a model. The methyl ester of (±)-alanine was subjected to the modified Clauson-Kaas pyrrole synthesis to give an α-pyrrolic ester **19** which was subjected to two carbon homologation by ester reduction with DIBAl-H followed by an *in situ* Wadsworth-Emmons olefination [18]. The alkene **20** was then hydrogenated to the γ-pyrrolic ester **21** and cyclised to give α-ketopyrrole **18** in 67% overall yield from **19**. The modified Knorr-Rabe reduction of **18** gave the desired pyrroline **22** in near quantitative yield as a 9:1 mixture of diastereomers. The volatility of the compound meant that for practical purposes it was isolated as the hydrochloride salt by adding concentrated HCl to the organic extract before evaporation. Catalytic hydrogenation of the hydrochloride salt of the pyrroline gave a corresponding mixture of isomers of 5-methylindolizidine **23** but to our surprise the *trans* isomer was the major diastereomer. The stereochemical assignment of the major and minor isomers was confirmed by comparison of the ^13^C NMR spectra with the reported spectra for both previously synthesised isomers [19]. The resonance of the carbon signals for C-8a, C-5 and C-3 are diagnostic with these carbons for the major isomer resonating 54.9, 50.2 and 49.1 ppm. This compares to 54.5, 50.0 and 49.2 ppm for the *trans* isomer and 64.8, 58.9 and 51.8 ppm for the *cis* isomer as reported in the literature [19]. This result indicates that the major product **22** from the modified Knorr-Rabe zinc reduction has the opposite C-5/C-8a stereochemistry to that typically obtained by catalytic hydrogenation.

To explain this result we propose that the zinc complexation to the less hindered face of the indolizidine causes protonation to occur on the same side as the C-5 substituent, which results in the *trans* stereochemistry between C-5 and C-8a.

**Scheme 8:**
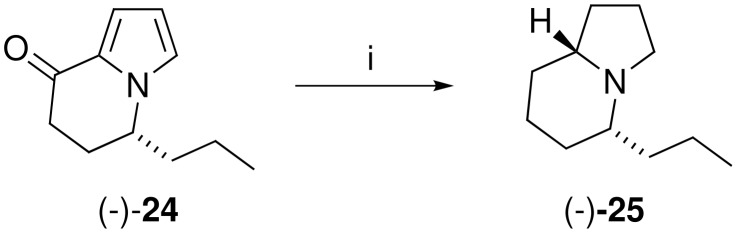
i) ref. [17].

**Scheme 9:**
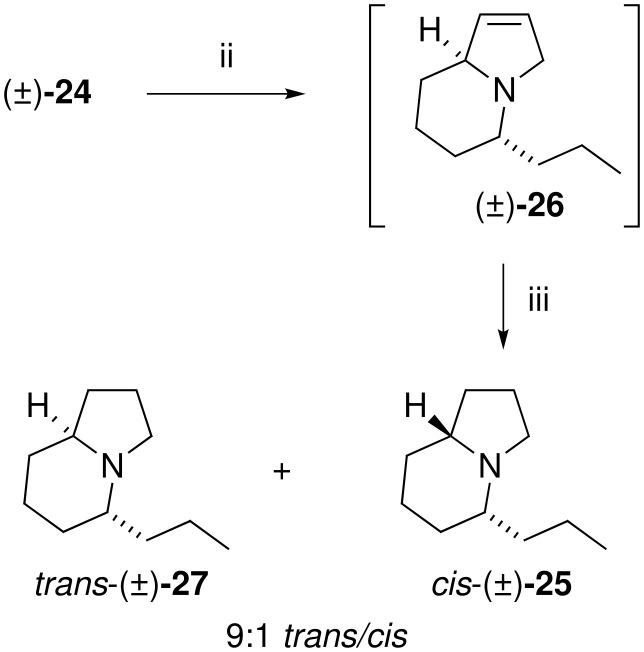
i) CH_3_OH, Zn, conc. HCl(aq) ii) H_2_ (40 psi), Pd/C, EtOH, 2M HCl.

A beneficial outcome from these observations is that one can now reduce bicyclic intermediates like **18** stereoselectively to enter either diastereomeric series. Corvo has reported the synthesis of the proposed structure of indolizidine 167B by the catalytic hydrogenation of (−)-**24** (Scheme 8) [17], and herein we report the racemic synthesis of its epimer (Scheme 9). We have reported the synthesis of the bicyclic ketone (±)-**24** [18] and subjection of this α-ketopyrrole to the modified Knorr-Rabe reduction conditions gave the crude 3-pyrroline **26** which was immediately subjected to catalytic hydrogenation to yield a 9:1 mixture of (±)-*epi*-indolizidine 167B (*trans*-(±)-**27**) and (±)-indolizidine 167B (*cis*-(±)-**25**) in 91% overall yield from **24**. As for the 5-methyl derivative the spectral data of the *trans* isomer **27** was dramatically different to that of the *cis* isomer **25** and is consistent with that reported previously [20]. Therefore, this method extends the flexibility of bicyclic pyrroles as intermediates for the synthesis of indolizidine alkaloids, as diastereomeric targets can be accessed simply by the choice of reagent system for reduction of the pyrrole nucleus.

## Conclusion

In conclusion, we have discovered a modified method for the Knorr-Rabe partial reduction of electron rich pyrroles which is effective for the reduction of bicyclic α-ketopyrroles to the corresponding 3-pyrroline or hexahydroindolizidine derivatives. The reduction occurs with high diastereoselectivity with 5-alkyl derivatives and gives the opposite diastereoselectivity to that of direct catalytic hydrogenation. This complementary method allows for the synthesis of both diastereomers of indolizidine 167B from a late-stage common intermediate.

## Supporting Information

File 1 Experimental details which includes experimental procedures and spectroscopic data
