# Supplementary material for: Knorr-Rabe partial reduction of pyrroles: Application to the synthesis of indolizidine alkaloids
Source: Beilstein J Org Chem. 2008 Jan 15;4:3. doi: 10.1186/1860-5397-4-3 (PMC2238744; doi:10.1186/1860-5397-4-3)
Supplement: File 1 — Experimental details which includes experimental procedures and spectroscopic data [file Beilstein_J_Org_Chem-04-03-s001.doc]

### Experimental details

### General

^1^H and ^13^C NMR spectra were recorded at 300 MHz and 75 MHz respectively on a Varian Mercury 2000 spectrometer. Spectra were run in CDCl_3_ unless otherwise stated. Chemical shifts were measured in ppm and referenced internally to residual CHCl_3_ for ^1^H NMR (7.26) and the central peak of CDCl_3_ for ^13^C NMR (77.16). Infrared spectra were recorded on a Perkin-Elmer FT-IR Paragon 1000 spectrometer as neat films on NaCl plates unless otherwise stated. Mass spectroscopy was performed on a Kratos Concept ISQ mass instrument. Analytical analyses were performed by The Central Science Laboratory at the University of Tasmania. Melting points were carried out on Yanagimoto Seisakusho micro melting point apparatus and are uncorrected. Flash chromatography was performed according to the method of Still and co-workers [1] using silica gel 60 (32-63μm). All solvents and reagents were available from Aldrich, AJAX or BDH chemicals and used as supplied or purified by standard laboratory methods if required. [2] Organic extracts were dried with anhydrous magnesium sulfate unless otherwise stated.

**3,5,6,7,8,8a-Hexahydroindolizidine (9)**

A stirred solution of 8-oxo-5,6,7,8-dihydroindolizine **8** [3] (0.12g, 0.89 mmol) in methanol (3 mL) was heated to reflux, then removed from the heat and powdered zinc (0.58 g, 8.9 mmol) and 10 M hydrochloric acid (3 mL) were added in small alternating portions to the reaction mixture over ~ 10 min. After the addition was complete the reaction mixture was cooled and made alkaline by addition of conc. ammonia (10 mL) then extracted with dichloromethane (3 x 10 mL). The dichloromethane extracts were dried (Na_2_SO_4_) and evaporated under reduced pressure until the solvent has just been removed to yield the volatile hexahydroindolizine **9** (~ 80% ) as an oil contaminated with a trace of solvent in.

^1^H NMR δ: 1.30 (m,2H), 1.57 (m, 2H), 1.80 (m, 2H), 2.48 (m, 1H), 3.00 (m, 2H), 3.15 (dd, *J* = 12 and 6 Hz, 1H), 3.58 (m, 1H), 5.84 (m, 2H). ^13^C NMR δ: 24.1, 24.7, 29.4, 49.9, 57.5, 67.3, 128.1, 133.6.

**Reduction of alcohol 11 to 3,5,6,7,8,8a-Hexahydroindolizidine (9)**

Sodium borohydride (14 mg, 0.37 mmol) was added to a stirred solution of the a-ketopyrrole **8** (50 mg, 0.37 mmol) in ethanol (2 mL) at ambient temperature followed by a second portion of sodium borohydride (14 mg, 0.37 mmol) after 5 min and the reaction monitored by TLC to follow the conversion of the ketone **8**  to the alcohol **11**. The alcohol **11** was then reacted *in-situ* by the addition of powdered zinc (240 mg, 3.7 mmol) and conc. HCl (3 mL) following the method reported above to give hexahydroindolizine **9** as the only product by ^1^H and ^13^C nmr.

**Reduction of pyrrole 13 to 3,5,6,7,8,8a-Hexahydroindolizidine (9)**

Pyrrole **13** was prepared from a-ketopyrrole **8** by the method of Vallée [4] and subjected to the reduction conditions reported above to yield impure hexahydroindolizine **9** as the major product as indicated by ^1^H and ^13^C nmr.

**(±)-*trans*-5-Methyl-3,5,6,7,8,8a-hexahydroindolizidine hydrochloride (22)**

A stirred solution of 5-methyl-8-oxo-5,6,7,8-dihydroindolizine **18** [5] (0.10 g, 0.67 mmol) in methanol (3 mL) was heated to reflux, then removed from the heat and powdered zinc (0.44 g, 6.7 mmol) and 10 M hydrochloric acid (3 mL) was added in small alternating portions to the reaction mixture over ~10 min. The reaction mixture was made alkaline with conc. ammonia (10 mL) and extracted with dichloromethane (3 x 10 mL). The dichloromethane extracts were combined and 10 M hydrochloric acid (2 drops) added. The reaction mixture was stirred for 15 h then evaporated under reduced pressure to yield the hydrochloride salt **22** (quantitative).

Major isomer *trans*-**23** ^1^H NMR δ: 1.42 (d, *J* = 6.3 Hz, 3H), 1.64 (m, 4H), 1.92 (m, 2H), 3.15 (m, 1H), 3.74 (m, 1H), 4.23 (m, 1H), 4.42 (m, 1H), 5.78 (m, 2H), 11.4 (bs, 1H). ^13^C NMR δ: 16.7, 17.4, 24.3, 27.3, 55.1, 57.4, 65.0, 124.3, 129.9. IR *ν*_MAX_: 3400, 2947, 2604, 2499, 1641, 1474, 1444, 1397, 1037, 807, 704 cm^-1^. MS (EI)*m/z*: 137(45%, M^+^) 136(50), 122(65), 94(100), 80(60). HRMS-EI *m/z*: 137.1205 [M-HCl]^+^ calcd for C_9_H_15_N: 137.1205.

**(±)-*trans*-5-Methyloctahydroindolizidine (23)**

A mixture of alkene hydrochloride **22** (56 mg, 0.32 mmol) and 10% Pd/C (20 mg) and 2M HCl (0.1 mL) in ethanol (5mL) was shaken vigorously under an atmosphere of hydrogen at 40 psi on a Parr shaker hydrogenator for 2 h. The hydrogenation mixture was filtered through Celite™, the filtrate evaporated to dryness and treated with 2M sodium bicarbonate (5 mL). The solution was extracted with dichloromethane (3 x 10 mL), the combined extracts were dried and concentrated to yield 5-methyloctahydroindolizidine **23** (41 mg, 92%) as a 9:1 mixture of *trans*/*cis* diastereomers. The spectral data are consistent with that previously reported. [6]

Major isomer *tran*-**23**: ^1^H NMR δ: 0.99 (d, *J* = 6.6Hz, 3H), 1.84-1.16 (m, 10H), 2.46-2.54 (m, 1H), 2.58 (q, *J* = 8.1Hz, 1H), 2.84 (td, J=9.3 and 3.0 Hz, 1H), 3.22 (m, 1H). ^13^C NMR δ: 10.4, 19.0, 20.7, 30.1, 30.9, 31.1, 49.1, 50.2, 54.9.

Minor isomer *cis*-**23**: ^13^C NMR δ: 20.3, 21.0, 24.7, 30.5, (31.1 obscured), 34.2, 51.7, 58.2, 64.8.

**(±)-*trans*-5-Propyloctahydroindolizidine (27)**

A stirred solution of 5-propyl-5,6,7,8-dihydroindolizine [5] **24** (57 mg, 0.32 mmol) in methanol (3 mL) was heated to reflux, then removed from the heat and powdered zinc (0.21 g, 3.22 mmol) and 10 M hydrochloric acid (2 mL) were added in small alternating portions to the reaction mixture over ~ 10 min . After the addition was complete the reaction mixture was made alkaline by addition of conc. ammonia (10 mL) and extracted with dichloromethane (3 x 10 mL). The dichloromethane extracts were combined and 10 M hydrochloric acid (2 drops) was added. The reaction mixture was then evaporated under reduced pressure to yield the hydrochloride salt **26**. A mixture of the crude hydrochloride **26**, 10% Pd/C (20 mg) and 2M HCl (0.1 mL) in ethanol (5mL) was shaken under an atmosphere of hydrogen at 40 psi on a Parr shaker hydrogenator for 2 h. The hydrogenation mixture was filtered through Celite™, evaporated to dryness and 2M sodium bicarbonate (5 mL) added. The solution was extracted with dichloromethane (3 x 10 mL), the combined extracts dried and concentrated to yield **27** as a clear oil (49 mg, 91%) as a 9:1 mixture of *trans*/*cis* diastereomers. The spectral data are consistent with that previously reported. [7]

Major isomer *trans*-**27**:  ^1^H NMR (major isomer) δ: 0.88 (t, *J* = 7.2Hz, 3H), 1.10 – 1.82 (m, 14H), 2.47 (m, 1H), 2.64 (q, *J* = 7.8, 1H), 2.91 (m, 1H), 2.82 (td, *J* =8.7 and 3.0 Hz, 1H), ^13^C NMR δ: 14.4, 19.2, 20.7, 20.8, 25.9, 27.4, 30.4, 30.9, 48.7, 55.30, 55.33.

Minor isomer *cis*-**25**: ^13^C NMR δ: 14.5, 19.1, 20.4, 24.7, 30.5, 30.8, (31.1 obscured), 36.8, 51.5, 63.7, 65.0.

**References**

1 Still WC, Kahn M, Mitra A: *J Org Chem* 1978, **43**:2923-2925.

2 Perrin DD, Armarego WLF, Perrin DR: Purification of Laboratory Chemicals. Oxford: Pergamon Press; 1980.

3 Amos R I J, Gourlay BS, Molesworth, PP, Smith JA, Sprod O R: *Tetrahedron* 2005, **61**: 8226 - 8230.

4 Sayah B, Pelloux-Léon N, Vallée Y: *J Org Chem* 2000, **65**: 2824-2826.

5 Gourlay BS, Molesworth PP, Ryan JH, Smith JA: *Tetrahedron Lett* 2006, **47**:799-801.

6 Tehrani KA, D’hooghe M, Kimpe ND: *Tetrahedron* 2003, **59**:3099-3108.

7 Polniaszek RP, Belmont SE: *J Org Chem* 1990, **55**: 4688-4693.
